# Relationship between continuity of care and adverse outcomes varies by number of chronic conditions among older adults with diabetes

**DOI:** 10.15256/joc.2016.6.76

**Published:** 2016-06-03

**Authors:** Eva H. DuGoff, Karen Bandeen-Roche, Gerard F. Anderson

**Affiliations:** ^1^Department of Population Health Sciences, School of Medicine and Public Health, University of Wisconsin-Madison, Madison, WI, USA; ^2^Department of Biostatistics, Bloomberg School of Public Health, Johns Hopkins University, Baltimore, MD, USA; ^3^Department of Health Policy and Management, Bloomberg School of Public Health, Johns Hopkins University, Baltimore, MD, USA

**Keywords:** Multimorbidity, continuity of patient care, care coordination, Medicare, patient complexity, chronic disease, multiple chronic conditions

## Abstract

**Background:**

Continuity of care is a basic tenant of primary care practice. However, the evidence on the importance of continuity of care for older adults with complex conditions is mixed.

**Objective:**

To assess the relationship between measurement of continuity of care, number of chronic conditions, and health outcomes.

**Design:**

We analyzed data from a cohort of 1,600 US older adults with diabetes and ≥1 other chronic condition in a private Medicare health plan from July 2010 to December 2011. Multivariate regression models were used to examine the association of baseline continuity (the first 6 months) and the composite outcome of any emergency room use or inpatient hospitalization occurring in the following 12-month period.

**Results:**

After adjusting for baseline covariates, high known provider continuity (KPC) was associated with an 84% (adjusted odds ratio 0.16; 95% confidence interval 0.09–0.26) reduction in the risk of the composite outcome. High KPC was significantly associated with a lower risk of the composite outcome among individuals with ≥6 conditions. However, the usual provider of care and continuity of care indices were not significantly related with the composite outcome in the overall sample or in those with ≥6 conditions.

**Conclusion:**

The relationship between continuity of care and adverse outcomes depends on the measure of continuity of care employed. High morbidity patients are more likely to benefit from continuity of care interventions as measured by the KPC, which measures the proportion of a patient’s visits that are with the same providers over time.

## Introduction

Older adults living with multiple chronic conditions (MCCs) are more likely to experience adverse events [1, 2] and the risk of these events increases with the number of chronic conditions [3]. Continuity of care is a basic tenant of primary care practice and leading expert groups in the USA and internationally have emphasized its importance for individuals with chronic diseases [4, 5]. The prevailing theory is that older adults with MCCs are more likely to experience adverse events due to healthcare system fragmentation resulting from a lack of continuity of care. Care coordination interventions are expected to improve continuity of care, with a coherent and connected experience of the healthcare system, and receipt of care that is consistent with the patient’s needs, leading to improved outcomes for older adults with MCCs [6]. However, while a few programs have shown promise [7, 8], most have reported null results [9, 10].

One reason past interventions may not have performed well is that our understanding of the association of continuity of care and adverse outcomes in the context of MCCs is limited. The few studies that have examined the association of continuity of care and adverse outcomes in populations with substantial chronic condition burden have reported conflicting results. A study of older adults in the US Medicare program, where half of the sample reported ≥3 chronic conditions, found that greater visit-based continuity of care was associated with greater risk of adverse outcomes [11]. Similarly, a study of individuals with chronic conditions in Taiwan reported similar results for physician-based continuity measures [12]. In contrast, other studies have found that higher continuity is associated with lower risk of emergency department use and inpatient hospitalization in older adults with ≥3 chronic conditions using an integrated healthcare delivery system and lower risk of hospitalization or death among US adults with diabetes [13, 14]. Older adults living with ≥6 chronic conditions are an important population to policymakers because they account for only 14% of Medicare’s enrollment but 43% of Medicare spending [15]; however, there are no studies examining continuity of care in this subpopulation. A better understanding of continuity of care in complex patients is necessary to design appropriate interventions to reduce adverse events. To this end, it is important to look at the measures themselves that are used to identify continuity of care.

There are several approaches to measuring continuity of care [16]. Researchers typically use visit-based measures of continuity to capture the concentration of a patient’s visits with a particular provider (e.g., usual provider of care [UPC] index) or the distribution of a patient’s visits across several providers (continuity of care [COC] index) [17, 18]. In the context of individuals with MCCs, however, these traditional measures may not accurately measure the continuity of care for individuals who see multiple physicians regularly. Researchers recently proposed a new measure of continuity of care, the known provider of care (KPC) index, which measures the proportion of a patient’s visits that are with the same providers over time [19]. The KPC index may be particularly relevant for older adults with MCCs who may have long-term relationships with several physicians for the management of their chronic conditions.

We study these issues in the context of a Medicare Advantage Chronic Care Special Needs Plan (SNPs). Congress created Medicare Advantage SNPs to incentivize private health plans to enroll high-cost, vulnerable beneficiaries who might benefit from specialized services and care coordination. Chronic Care SNPs are a type of SNP that focuses on Medicare beneficiaries with specific severe or disabling chronic conditions such as diabetes. Other SNPs focus on individuals dually eligible for Medicare and Medicaid, and people who are institutionalized. Studying this population provided an opportunity to examine the relationship between continuity of care and adverse outcomes among those who we expect to most likely benefit from greater continuity.

## Objective

The aim of the study was to examine the relationship between continuity of care and adverse outcomes as measured by emergency room and inpatient hospitalization utilization. We compared three measures of continuity of care: concentration of care (UPC), dispersion of care (COC), and relational continuity (KPC). As the number of chronic conditions is strongly related to adverse outcomes, we also examined how the relationship between continuity of care and adverse outcomes varies by the number of chronic conditions for each measure of continuity.

## Materials and methods

### Study data

This is a retrospective study of US older adults with diabetes and at least one other chronic condition enrolled in a Medicare Advantage Chronic Care SNP. The study used administrative claims and care management data from July 1, 2010 to December 31, 2011 from 1,600 subjects selected to participate in a patient mail survey. The first 6 months (July–December 2010) was the baseline period, and the following 12 months (January–December 2011) was the follow-up period.

Survey subjects were selected based on the following criteria: age ≥65 years as of July 1, 2010; enrolled in one of the Chronic Care SNP’s Preferred Provider Organization plans, which were available in Arkansas, Georgia, Missouri, South Carolina, and Texas; and identified as having diabetes and at least one other chronic condition. We excluded 52 subjects who died and 90 who disenrolled from the SNP during the study period.

The study sample was selected using stratified random sampling based on the subject’s number of chronic conditions. Chronic condition counts were constructed for the sampling frame using the Agency for Healthcare Research and Quality (AHRQ) Clinical Classification Software and Chronic Condition Indicator, which identified up to 190 different chronic conditions using claims data from July 1, 2010 to March 30, 2011 [20, 21]. Individuals were grouped into five strata according to the number of observed chronic conditions (<3, 3–4, 5–6, 7–8, ≥9). This approach ensured sufficient sample size of individuals with MCCs for subgroup analyses of the highest cost Medicare beneficiaries [15].

The Johns Hopkins Bloomberg School of Public Health and University of Wisconsin-Madison Institutional Review Boards approved this study.

### Continuity of care

We measured continuity of care using three measures: the UPC, COC, and KPC indices. We used previously published algorithms to construct these measures (see Supplementary Table 1). We briefly describe each measure below.

The UPC quantifies the proportion of a patient’s physician visits with the physician who provided the plurality of care. The COC measures the dispersion of a patient’s care across all providers. The UPC and COC indices are two of the most commonly used measures of visit-based continuity [17, 22]. The UPC and COC indices were constructed using data from the baseline period (July–December 2010). The KPC quantified the proportion of physicians seen during the baseline period (July–December 2010) who were also seen in the follow-up period (January–December 2011) [19]. All continuity measures were constructed on a 0–1 scale (Supplementary Figure 1). We categorized each measure into tertiles based on the sample-weighted distribution.

Older adults with MCCs see multiple primary care and specialist physicians [23], and it is likely that in some situations a specialist physician may be the patient’s primary care provider. Therefore, we constructed continuity measures using all primary care and specialist physician visits excluding physician specialties with limited patient interactions such as anesthesiologists and pathologists. A physician visit was defined as an outpatient medical bill with a date of service using the Berenson-Eggers Type of Service Codes, “evaluation and management” and “visits for procedures”, with a relative value ≥2.0 [24].

Claims-based measures are unstable when measured with few physician visits. For example, a person with two visits to the same physician will have a UPC score of 1, but a person with one visit to two different providers will have a UPC score of 0.5. For this reason we excluded 204 subjects with ≥3 visits in the baseline period.

### Covariate measures

We identified relevant covariates using the Aday-Andersen health behavior model [25]. We measured age as a categorical variable (65–70, 71–80, and >80 years), gender, race (White and not White), and state of residence. As a proxy for income, we used the presence of any Medicaid enrollment during the baseline period. Familiarity with a health plan’s processes and procedures may also promote access to primary care and reduce emergency room use, and so we accounted for time enrolled in the health plan (<1 year and ≥1 year).

The number of chronic conditions was measured with the Clinical Classification Software and Chronic Condition Indicator using claims for the 18-month study period [20, 21]. We used all available data to determine the number of chronic conditions because administrative data often underreport their presence [26].

We used baseline physician visits (<5, 5–10, and ≥11) to account for any residual differences among high and lower service utilizers. We also accounted for the occurrence of the outcome of interest during the baseline period.

### Outcome measures

The primary outcome of interest was the composite measure of any emergency room visit or any inpatient hospitalization occurring during calendar year 2011. We identified emergency room visits from claims data using Current Procedural Terminology codes 99281–99285 and dates of service [27]. Hospitalizations were identified using dates of service and place of service codes. We categorized each indicator as binary, where 0 represented no events during the calendar year and 1 presented one or more events.

### Analytic approach

All analyses were conducted in RStudio [28]. Sampling weights were calculated as the inverse probability of being selected to be in the sample. We used Chi-square tests to assess the bivariate differences between groups, and sample-weighted logistic regression models to examine the multivariate association of continuity during the baseline period and adverse health events occurring in calendar year 2011. We examined the correlation of continuity measures using Spearman correlation coefficients.

Separate multivariate logistic regression models were used to examine the association of UPC, COC, and KPC and a composite adverse outcome (any emergency room visits or any inpatient hospitalization) occurring during the 12-month follow-up period. To assess for variation by number of chronic conditions, we ran separate models examining individuals with <6 conditions and those with ≥6 chronic conditions. All models accounted for patient level factors including number of chronic conditions, gender, race, Medicaid status, duration enrolled in the health plan, and state of residence.

We report the adjusted odds ratios and 95% confidence intervals (CI) for the main effect. CIs were constructed as exp(log(odds β) ± 1.96 × standard error). Because the continuity measures are categorized into tertiles comparing high to low and middle to low using separate indicators, we also examined the joint significance of both indicators using the Wald test.

We conducted several sensitivity tests. We examined the association when the dependent variable was specified as a count of events. We also examined the sensitivity of our results to the specification of the model covariates. We tested models where UPC and COC were measured using only primary care providers, and UPC, COC, and KPC were treated as continuous and categorized as binary at 0.5 and quartiles. While six chronic conditions is a subgroup important to US policymakers because it identifies a high-cost group, we also examined groups with a greater chronic condition burden [15].

## Results

The data from 1,254 older adults with MCCs were eligible for the study. A majority of subjects were female, racial minorities, and enrolled in Medicaid (Table 1). Over 70% of subjects were enrolled in the health plan for ≥1 year. The average number of chronic conditions in the sample was 7.6; 15% of the sample had <6 chronic conditions and 84% had ≥6 chronic conditions. Individuals with <6 chronic conditions were less likely to be enrolled in Medicaid (41% vs. 57%; *p*<0.001) and less likely to experience an emergency room visit (22% vs. 45%; *p*<0.001) or inpatient hospitalization (12% vs. 23%; *p*<0.001).

Table 2 presents the pair-wise correlation of continuity of care measures overall and by number of chronic conditions. In the overall sample, the UPC and COC measures were highly correlated (*r*=0.86), and the KPC measure was weakly correlated with both the UPC and COC measures (*r*<0.3).

Greater visit-based continuity, as measured by the UPC, COC, and KPC indices, was associated with lower odds of any adverse outcome in unadjusted models (Table 3). After adjusting for patient level factors including number of chronic conditions and occurrence of any adverse event during the baseline period, only KPC continuity was significantly associated with the composite outcome of emergency room visit or inpatient hospitalization (*p*<0.001, Wald test). In adjusted models, high versus low KPC continuity was associated with an 84% reduction in the risk of the composite outcome (adjusted odds ratio [aOR] 0.16; 95% CI 0.09–0.26).

Table 4 presents the subgroup analyses by number of chronic conditions. Among individuals with <6 chronic conditions, the odds of the composite outcome did not significantly differ between those with high and middle UPC, COC, and KPC continuity and low levels of continuity. However, KPC continuity significantly improved model fit (*p*=0.002, Wald test). Among those with ≥6 chronic conditions, the odds of an adverse event decreased by 86% (aOR 0.14, 95% CI 0.08–0.25) in those with high compared to low KPC continuity, and decreased by 49% (aOR 0.51, 95% CI 0.29–0.93) in those with middle compared to low KPC continuity. UPC and COC were not significantly related to the composite outcome among older adults with ≥6 chronic conditions.

We ran several sensitivity tests to assess the robustness of our results. Higher levels of KPC continuity were associated with lower odds of the composite outcome in analyses using counts of adverse events (Supplementary Table 2) and alternative specifications of continuity (Supplementary Table 3). We tested alternative thresholds for the subgroup analyses at ≥8 and ≥10 chronic conditions (Supplementary Table 4). In these subgroups, higher levels of KPC continued to be significantly associated with lower odds of the composite outcome. In contrast to the main analysis, high versus low COC continuity was associated with three times greater odds (aOR 2.99; 95% CI 1.44–6.20) of the composite outcome among older adults with ≥8 chronic conditions, and 5.3 times greater odds (aOR 5.28; 95% CI 1.72–16.25) in those with ≥10 chronic conditions.

## Discussion

In a sample of older adults living with diabetes and at least one other chronic condition, we found that high continuity of care, as measured by the KPC, is associated with lower odds of experiencing an emergency room visit or inpatient hospitalization. We also found that the benefits of high KPC continuity were greater among older adults with ≥6 chronic conditions than in those with <6 conditions. We were surprised to find that commonly used measures of continuity, the UPC and COC indices, were not associated with adverse events in the overall sample or primary subgroup analysis.

This study found substantially lower emergency room and hospital use among older adults with MCCs seeing the same physicians over time. This result supports the hypothesis that continuity of care is important for older adults with MCCs and reinforces the importance of relational continuity in this population. However, the study also shows that the commonly used claims-based measures of continuity of care – the UPC and COC indices –were not predictive of adverse health outcomes. The reason may be that point-in-time estimates of a patient’s physician team and care patterns are not adequate in this population. While focusing on care patterns may be appropriate for individuals with a new diagnosis or those who are relatively healthy, older adults with MCCs may have substantially different health needs and their care teams may be naturally and appropriately diffuse.

We also found that the KPC correlated weakly with the UPC and COC. The high correlation between the UPC and COC is consistent with previous studies [29, 30]. This is the first study to compare the KPC to these standard measures. While the KPC also relies on claims data for its construction, its focus on which providers are seen over time may explain why it is so poorly correlated with the UPC and COC. These results suggest that the KPC index is a fundamentally different construct from the UPC and COC indices.

Previous studies of continuity of care and adverse outcomes have generally reported that greater continuity was associated with lower risk of adverse events in children, older adults generally, and even those with selected conditions [22, 31–33]. However, the evidence on continuity of care in the context of MCCs is mixed. Reports indicate that greater continuity was associated with lower risk of adverse events among older adults in an integrated healthcare delivery system [13], and among working aged adults enrolled in a US private health plan [14]. In contrast, greater continuity was associated with higher risk of adverse events among older adults enrolled in the fee-for-service US Medicare program and among individuals with chronic conditions in Taiwan [11, 12]. This study fills an important gap in the current evidence by examining whether the relationship between continuity of care and adverse outcomes varies by number of chronic conditions, and uses a novel measure of continuity to understand what measure of continuity of care may be most predictive in a population with MCCs.

We know that individuals with MCCs are heterogeneous in their conditions and level of complexity. We found that continuity in one’s providers over time (KPC) was associated with lower risk of adverse outcomes overall, and these benefits were greater among those with ≥6 chronic conditions.

Subgroup analyses examining individuals with ≥6, ≥8, and ≥10 chronic conditions yielded surprising results. While the relationships between the UPC and COC and the composite outcome were not significant among older adults with ≥6 chronic conditions, the coefficients were in the opposite direction than expected. Among individuals with a greater chronic condition burden, high COC continuity was associated with greater odds of an emergency room visit or inpatient hospitalization compared to low continuity. The relationship between the UPC and composite outcome was also positive, but not significant. One explanation is that these claims-based measures of continuity may not capture the full range of services needed by the most complex patients. Alternatively, claims-based COC measures may be subject to complex and time-varying confounding by health status, such that high COC more accurately reflects impending health declines than it does continuity of care due to either the anomalies of claims-based measurement or reverse effects by which health changes affect the nature of healthcare. It is also possible that, among older adults with substantial complexity, having many different doctors may be appropriate as long as the patient sees them with sufficient frequency.

We found that this SNP population was markedly less healthy than the general Medicare population. While 14% of the Medicare fee-for-service population is living with ≥6 chronic conditions [15], we found a corresponding value of over 80% in this study sample. These results are consistent with industry reports that the SNPs generally have higher risk scores than the Medicare fee-for-service program [34].

These results may have important implications for healthcare providers and quality measure developers interested in improving continuity of care for older adults with MCCs. Measures of continuity of care are being used to evaluate care coordination interventions and to monitor care coordination programs. The National Committee for Quality Assurance, for example, requires patient-centered medical homes to monitor the proportion of a patient’s visits with his or her provider, a measure very similar to the UPC [35]. These study results suggest that visit-based continuity measures focusing on the concentration of care with a set of providers or primary provider may not be appropriate for the most complex patients. Measures that capture the consistency in a patient’s providers over time may be more informative of continuity, especially for individuals with substantial numbers of chronic conditions.

This study has several strengths. It draws on a sample of older adults who were purposefully selected by number of chronic conditions to allow for analyses among individuals living with substantial complexity. This selection strategy provided sufficient sample size to assess subgroups of older adults with ≥6, ≥8, and ≥10 chronic conditions. We tested commonly used measures of continuity and alternative specifications that are used widely in the literature, and also examined a new measure of continuity. This study draws on individuals with diabetes, a common chronic condition that is associated with substantial morbidity and mortality in the USA and internationally. Lastly, this study uses a pre-post study design to overcome limitations common in the cross-sectional studies of continuity.

Several study limitations should be noted. This study uses administrative measures of continuity of care, which do not capture information sharing between clinicians or appropriate referrals to specialists. In addition, the plurality provider identified in the 6-month baseline period may not actually be the patient’s primary physician. There are several other continuity of care measures in the literature [16]. While previous studies have found high correlation among claims-based continuity measures, it is possible that alternative measures could lead to different results [29, 30]. We used all-cause hospitalization as an outcome but, of course, not all hospitalization events can be prevented with better continuity of care.

It is important to note that the study sample is not representative of the US Medicare fee-for-service population and may be substantially different from the general Medicare Advantage population. While these data are somewhat dated, we believe that the findings are still relevant because older adults with MCCs continue to see multiple physicians resulting in complex care patterns.

## Conclusions

Older adults with MCCs are frequent users of the healthcare system seeing multiple primary care and specialist physicians. Commonly used measures of continuity for the concentration of care (UPC) with a particular provider or dispersion of care (COC) across a set of providers may not capture important aspects of continuity in these patients. We found that, in older adults with MCCs, greater continuity of care as measured by the KPC, which quantifies the extent to which patients see the same providers over time, was associated with better outcomes. The results suggest that healthcare providers and healthcare systems should design care coordination interventions that encourage stable, long-term relationships between older adults with MCCs and their providers, and continuity of care measures should reflect the importance of relational continuity.

## Figures and Tables

**Table 1:** Baseline sample characteristics: overall and stratified by number of chronic conditions.

	Total	Chronic conditions		*p*
		<6	≥6	
Sample *n*	1,254	198 (15.8%)	1,056 (84.2%)	
Age (%)				0.434
65–70 years	29.8	31.7	28.9	
71–80 years	50.7	47.1	52.5	
≥80 years	19.5	21.3	18.6	
Sex, female (%)	66.2	67.0	65.8	0.774
Race, not white (%)	58.1	53.1	60.6	0.073
Enrolled ≥1 year (%)	69.9	69.4	70.1	0.856
Any Medicaid (%)	51.3	40.8	56.8	<0.001
Chronic conditions, mean (SD)	7.6 (3.6)	4.0 (0.9)	9.4 (3.1)	<0.001
Baseline utilization				
Physician visits (%)				<0.001
<6	38.7	61.0	27.6	
6–10	36.7	35.5	39.2	
≥11	24.7	7.5	33.2	
Any inpatient hospitalization (%)	19.4	12.4	22.9	<0.001
Any emergency room use (%)	37.1	22.3	44.5	<0.001
Composite adverse outcome (%)	42.2	27.8	49.4	<0.001

**Table 2:** Spearman correlation of continuity of care measures: overall and stratified by number of chronic conditions.

	UPC	COC	KPC
Overall			
UPC	1.0	0.86	0.11
COC		1.0	0.17
KPC			1.0
<6 Chronic conditions			
UPC	1.0	0.80	0.11
COC		1.0	0.14
KPC			1.0
≥6 Chronic conditions			
UPC	1.0	0.85	0.05
COC		1.0	0.13
KPC			1.0

COC, continuity of care index; KPC, known provider of care index; UPC, usual provider of care index.

**Table 3:** Unadjusted and adjusted^*^ association of continuity and composite measures of adverse outcomes.

	Unadjusted odds ratio (95% CI)	*p*	Adjusted odds ratio (95% CI)	*p*
UPC				
Low (Ref)	1.0		1.0	
Middle	0.65 (0.46–0.93)	0.019	1.13 (0.75–1.71)	0.545
High	0.54 (0.37–0.79)	0.002	1.18 (0.73–1.89)	0.495
Wald test		0.004		0.755
COC				
Low (Ref)	1.0		1.0	
Middle	0.68 (0.48–0.96)	0.029	1.05 (0.7–1.56)	0.819
High	0.53 (0.35–0.80)	0.002	1.07 (0.66–1.74)	0.776
Wald test		0.006		0.956
KPC				
Low (Ref)	1.0		1.0	
Middle	0.53 (0.33–0.85)	0.009	0.61 (0.36–1.01)	0.057
High	0.11 (0.07–0.18)	<0.001	0.16 (0.09–0.26)	<0.001
Wald test		<0.001		<0.001

^*^Models also adjusted for baseline age, race, sex, morbidity, plan enrollment duration, physician visits, occurrence of the adverse event during the baseline period, and state of residence. CI, confidence interval; COC, continuity of care index; KPC, known provider of care index; Ref, reference; UPC, usual provider of care index.

**Table 4:** Adjusted^*^ association of continuity and composite measures of adverse outcomes stratified by number of chronic conditions.

	<6 Chronic conditions		≥6 Chronic conditions	
	Adjusted odds ratio (95% CI)	*p*	Adjusted odds ratio (95% CI)	*p*
UPC				
Low (Ref)	1.0		1.0	
Middle	1.40 (0.49–3.95)	0.530	1.30 (0.83–2.04)	0.257
High	1.51 (0.56–4.10)	0.417	1.46 (0.85–2.51)	0.175
Wald test		0.711		0.340
COC				
Low (Ref)	1.0		1.0	
Middle	0.93 (0.40–2.19)	0.869	1.35 (0.85–2.15)	0.202
High	0.85 (0.33–2.24)	0.750	1.49 (0.85–2.60)	0.162
Wald test		0.950		0.305
KPC				
Low (Ref)	1.0		1.0	
Middle	1.81 (0.42–7.83)	0.429	0.51 (0.29–0.93)	0.027
High	0.30 (0.08–1.12)	0.075	0.14 (0.08–0.25)	<0.001
Wald test		0.002		0.003

^*^Models also adjusted for baseline age, race, sex, number of chronic conditions, plan enrollment duration, physician visits, occurrence of the adverse event during the baseline period, and state of residence. CI, confidence interval; COC, continuity of care index; KPC, known provider of care index; Ref, reference; UPC, usual provider of care index.
